# CLR (CRP to Lymphocytes) Score for Differentiating Simple and Complicated Appendicitis in Pediatric Patients

**DOI:** 10.3390/jcm15010393

**Published:** 2026-01-05

**Authors:** Adir Alper, Ariel Galor, Mathias Lerner, Omer Levy, Osnat Zmora

**Affiliations:** 1 Shamir Medical Center, Be’er Ya’acov 60930, Israel; alpera@nychhc.org (A.A.);; 2 Department of Pediatric Surgery, Shamir Medical Center, Be’er Ya’acov 60930, Israel

**Keywords:** acute appendicitis, biomarkers, complicated appendicitis, CRP-to-lymphocyte ratio, CLR, NLR, PIV, MLR, PLR

## Abstract

**Background**: Acute appendicitis, a frequent pediatric surgical emergency, requires distinguishing simple from complicated cases for treatment decisions. Current tools, such as clinical scores and ultrasound, are sometimes ineffective. This study evaluates the biomarkers: neutrophils to lymphocytes ratio (NLR), monocytes to lymphocytes ratio (MLR), platelet-to-lymphocyte ratio (PLR), neutrophils to monocytes ratio (NMR), neutrophils to platelet ratio (NPR), pan-immune-inflammation value (PIV) ratio, and C-Reactive Protein (CRP) to lymphocytes ratio (CLR) for differentiation between simple and complicated appendicitis. **Methods**: A retrospective study of 878 pediatric patients (<18 years) who underwent appendectomy (2018–2024) at a tertiary medical center, with appendicitis classified as simple (SA, *n* = 696) or complicated (CA, *n* = 182) using intraoperative findings. Biomarkers were calculated from preoperative blood counts and CRP. Diagnostic accuracy was assessed using Mann–Whitney U tests, ROC curves, and logarithmic regression. **Results**: Patients with CA had higher neutrophils counts (13.61 ± 4.92 vs. 11.39 ± 4.29 K/μL), monocytes counts (1.23 ± 1.41 vs. 0.95 ± 0.48 K/μL), platelet counts (294.31 ± 72.73 vs. 270.15 ± 72.08 K/μL), CRP levels (88.55 ± 97.75 vs. 27.15 ± 44.74 mg/L), and elevated biomarker ratios as compared to those with SA: NLR (≥10.15, OR = 2.45), MLR (≥0.645, OR = 2.78), PLR (≥224.38, OR = 2.502), NMR (≥6.38, OR = 2.34), NPR (≥0.0405, OR = 1.876), PIV (≥2433.85, OR = 3.348), and CLR (≥11.77, OR = 5.935), all at *p* < 0.01. CLR demonstrated the highest accuracy (AUC = 0.772, sensitivity 78%, specificity 62.6%), outperforming established biomarkers, followed by PIV (AUC = 0.679). NPR was the least effective marker (AUC = 0.569). **Conclusions**: CLR, a promising biomarker, can aid in distinguishing complicated from simple appendicitis in children, and may offer accessible tools for resource-limited settings.

## 1. Introduction

Acute appendicitis is the most common surgical emergency in children, characterized by inflammation of the appendix that can progress from simple (uncomplicated) inflammation to complicated forms involving gangrene and/or perforation with generalized peritonitis or abscess formation. Early and accurate classification of pediatric appendicitis into simple versus complicated is crucial as it directly influences treatment decisions and patient outcomes. Simple appendicitis may be managed conservatively with antibiotics versus surgery within 24 h, while complicated appendicitis typically requires urgent surgical intervention and more intensive postoperative care for generalized peritonitis, as well as more prolonged intra-venous (IV) treatment and possible drainage for periappendicular abscesses. Misclassification or delayed diagnosis, especially in young children who often present with atypical symptoms, increases the risk of complications and associated morbidity [1,2].

Traditional diagnostic approaches, including clinical scores, ultrasonography, computed tomography (CT), C-Reactive Protein (CRP), and white blood cell (WBC) counts carry relatively low diagnostic accuracy [3]. Inflammatory biomarkers such as the neutrophil-to-lymphocyte ratio (NLR), platelet-to-lymphocyte ratio (PLR), and monocyte-to-lymphocyte ratio (MLR) have gained attention for their utility in diverse clinical settings, such as assessing the magnitude of surgical trauma and predicting postoperative complications. Elevated NLR levels, in particular, have been shown to correlate with the invasiveness of surgical procedures and may serve as early indicators of adverse outcomes, including infections and delayed recovery. Moreover, blood biomarkers such as NLR and PLR, have been used to predict the severity and prognosis of conditions including urinary tract infections, sepsis, and systemic inflammatory response syndrome (SIRS) [4,5].

Recent research has demonstrated the diagnostic ability of NLR, PLR, and MLR to distinguish between true acute appendicitis and a “white” appendix [6,7]. Prior meta-analyses have evaluated hematological parameters such as red cell distribution width (RDW) and mean platelet volume (MPV) as potential diagnostic markers for acute appendicitis [8,9]. Some studies even demonstrated the utility of such biomarkers in distinguishing complicated from simple appendicitis. For example, a 2023 study found that both NLR and PLR values were effective in detecting complicated appendicitis in children, supporting their use as adjuncts in clinical decision-making [10]. Similarly, a 2023 review highlighted that as the severity of appendiceal inflammation increases, NLR rises due to neutrophilia and lymphopenia, and the higher NLR values have been associated with gangrenous or perforated appendicitis [11]. In addition, a 2024 pediatric study demonstrated that inflammatory biomarkers such as NLR, PLR, and MLR were significantly higher in children with complicated appendicitis compared to those with uncomplicated disease, underscoring their role as indicators of increasing disease severity [12]. CLR was also reported by few small- and medium-scale studies to be effective in discriminating perforated from non-perforated appendicitis, as determined by histopathological examination in children and adults [13,14]. Even hyperbilirubinemia and hyponatremia have been proposed as indirect markers for complicated pediatric appendicitis (via bacterial translocation and inflammation-driven ADH release, respectively). A 2022 meta-analysis demonstrated hyperbilirubinemia with modest accuracy (sensitivity 62%, specificity 81%), while hyponatremia was associated with significantly lower serum sodium levels, on average 3.29 mmol/L lower in patients with complicated appendicitis [15,16]. Still, both suffered extreme heterogeneity (I^2^ = 98%), inconsistent cut-offs, and multiple confounders (liver disease, hemolysis, dehydration, vomiting, SIADH), limiting clinical utility.

In this study, we aimed to evaluate and compare multiple biomarkers in a large cohort of simple versus complicated pediatric appendicitis cases, as determined by operative findings, for more accurate and accessible tools in clinical treatment decisions.

## 2. Materials and Methods

### 2.1. Study Design and Population

We conducted a retrospective study of pediatric patients (aged < 18 years) who underwent appendectomy at our tertiary medical center from January 2018 to July 2024. The preoperative diagnosis of appendicitis was made by pediatric surgeons based on a combination of clinical signs and symptoms such as abdominal pain migrating to the right lower quadrant associated with nausea, anorexia, rebound tenderness, and guarding; laboratory results, such as elevated CRP and WBC counts; and ultrasound findings. The study included children with appendicitis of varying severity, ranging from early-stage simple appendicitis to complicated cases with gangrene, perforation, and abscess formation. The study was approved by the Shamir Medical Center Institutional Review Board (IRB), ensuring it met all ethical requirements.

### 2.2. Inclusion and Exclusion Criteria

Included were patients with complete preoperative blood counts and histopathology results. Excluded were pregnant patients, those with chronic inflammatory or immunologic diseases (e.g., inflammatory bowel disease), patients who underwent elective interval appendectomy, and patients with incomplete data (missing imaging or laboratory values).

### 2.3. Data Collection

Patients with histopathologically confirmed acute appendicitis were identified, then data collected on age, sex, temperature on admission, and the first laboratory results obtained in the emergency department at initial presentation, including complete blood counts (CBC) with differential (leukocytes, platelets, neutrophils, lymphocytes, monocytes), CRP levels, ultrasound findings, intraoperative findings, and histopathology reports. A senior pediatric surgeon classified cases using the American Association for the Surgery of Trauma (AAST) grading system into simple appendicitis (AAST grade 1) or complicated appendicitis (AAST grades ≥ 2, indicating gangrene, perforation, abscess, or peritonitis) based on intraoperative findings [17]. Intraoperative findings consistent with complicated appendicitis were: macroscopic gangrene, macroscopic opening in the appendiceal wall, free intraperitoneal pus, free fecolith, or a walled abscess.

### 2.4. Biomarker Calculations

Biomarkers were derived from CBC results (Sysmex XN-1000 analyzer, Kobe, Japan):

NLR = Neutrophils (K/μL)/Lymphocytes (K/μL)

MLR = Monocytes (K/μL)/Lymphocytes (K/μL)

NMR = Neutrophils (K/μL)/Monocytes (K/μL)

PLR = Platelets (K/μL)/Lymphocytes (K/μL)

NPR = Neutrophils (K/μL)/Platelets (K/μL)

PIV = Neutrophils (K/μL) × Monocytes (K/μL) × Platelets (K/μL) ÷ Lymphocytes (K/μL)

CLR = C-Reactive Protein (mg/L) ÷ Lymphocytes (K/μL).

### 2.5. Outcomes

Primary outcome: Diagnostic accuracy (AUC, sensitivity, specificity) of CLR for differentiating complicated (AAST ≥ 2) from simple (AAST grade 1) appendicitis.

Secondary outcomes: Diagnostic accuracy of NLR, MLR, PLR, NMR, NPR, and PIV, with comparison to CLR (AUC, sensitivity, specificity).

### 2.6. Statistical Analysis

Statistical analyses were conducted to examine the relationship between blood biomarkers and severity of appendicitis. Prior to selecting the appropriate tests, data normality was assessed using the Kolmogorov–Smirnov test and Q-Q plot. Depending on distribution and group size, comparisons between groups were performed using either parametric analysis (T-test) or non-parametric (Mann–Whitney U test). A *p*-value ≤ 0.05 was considered significant. To evaluate the diagnostic accuracy of various biomarkers, receiver operating characteristic (ROC) curves were generated, and the area under the curve (AUC) was calculated. Optimal cut-off values for all biomarkers were determined using the Youden index (J = sensitivity + specificity − 1) to maximize combined sensitivity and specificity. Outliers were inspected using the IQR method; no values were excluded if they were deemed biologically plausible. Sensitivity, specificity, positive predictive value (PPV), negative predictive value (NPV), and odds ratios (OR) with 95% confidence intervals were calculated. ORs were obtained by cross-tabulation and binary logistic regression. The dependent variable was appendicitis classification (complicated = 1, simple = 0), with independent variables including age, CRP, temperature, and binary variables for CLR (>11.77) and PIV (>2433.85). Odds ratios with 95% confidence intervals were calculated, and a *p*-value ≤ 0.05 was considered significant. Model fit was assessed using the Hosmer-Lemeshow test. Confidence intervals for sensitivity and specificity were calculated using Wilson score method based on 2 × 2 contingency tables.

All statistical analyses were performed using IBM SPSS Statistics v27.0 (IBM Corp., Armonk, NY, USA).

## 3. Results

**Figure 1:**
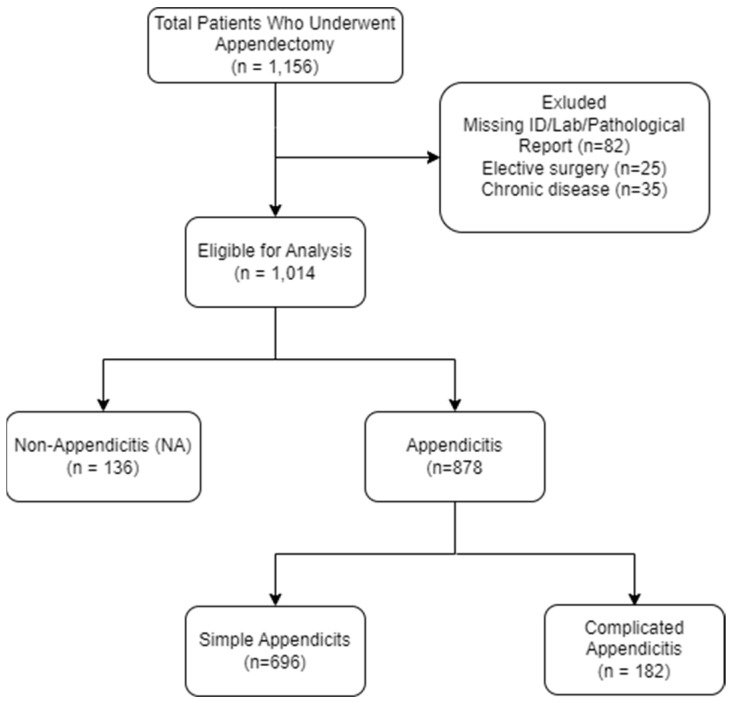
Flow Diagram of Study Population.

After exclusion of patients according to the exclusion criteria, 878 pediatric patients, who underwent appendectomy at Shamir Medical Center from January 2018 to July 2024, were included in the study. Of note, missing biomarker data affected < 2% of records; these patients were excluded and did not differ significantly in age, sex, or disease severity from the included cohort. Of these, 696 (79.3%) were diagnosed with simple appendicitis (SA), and 182 (20.7%) with complicated appendicitis (CA) (Figure 1).

When comparing children with SA to those with CA (Table 1), sex was found to be comparable between the two groups (*p* = 0.11). CA was associated with younger age (mean 10.0 ± 4.26 years compared to 11.8 ± 3.71 years in children with SA, *p* < 0.001).

Preoperative laboratory parameters revealed significant differences between the SA and CA groups. Patients with CA exhibited higher mean neutrophil counts (13.61 ± 4.92 K/μL vs. 11.39 ± 4.29 K/μL; *p* < 0.001), monocyte counts (1.23 ± 1.41 K/μL vs. 0.95 ± 0.48 K/μL; *p* < 0.001), platelet counts (294.31 ± 72.73 K/μL vs. 270.15 ± 72.08 K/μL; *p* = 0.031), and CRP levels (88.55 ± 97.75 mg/L vs. 27.15 ± 44.74 mg/L; *p* = 0.001) compared to those with SA. Conversely, lymphocyte counts were lower in the CA group (1.50 ± 0.86 K/μL vs. 1.74 ± 0.85 K/μL; *p* < 0.001). Body temperature demonstrated a statistically significant but clinically minor difference (37.27 ± 0.74 °C vs. 36.98 ± 0.45 °C; *p* < 0.001) (Table 1).

The CA group demonstrated significantly higher levels of all inflammatory biomarkers compared to the SA group: NLR (12.19 vs. 8.89; *p* < 0.001), MLR (1.02 vs. 0.68; *p* < 0.001), PLR (254.89 vs. 194.94; *p* < 0.001), NMR (12.23 vs. 8.90; *p* < 0.001), NPR (0.048 vs. 0.044; *p* = 0.004), PIV (4558.18 vs. 2393.29; *p* < 0.001), and CLR (81.73 vs. 20.03; *p* < 0.001) (Table 1).

**Figure 2:**
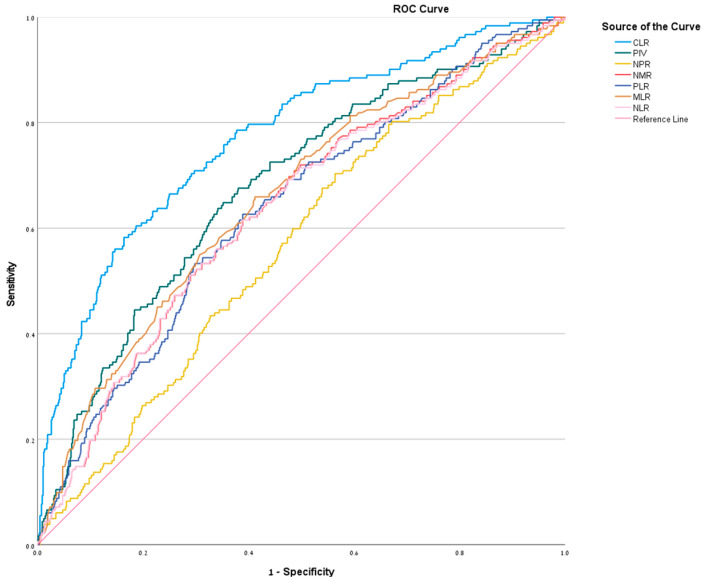
ROC curve for NLR, MLR, PLR, NMR, NPR, PIV, and CLR—illustrates graphically the relationship between true-positive rate (sensitivity) and false-positive rate (1-specificity).

ROC analysis was performed to evaluate the diagnostic performance of each biomarker in distinguishing CA from SA. CLR exhibited the highest discriminatory ability, with an AUC of 0.772 (95% CI: 0.733–0.812), achieving a sensitivity of 77% and specificity of 62.6% at a cutoff of ≥11.77. The PIV ranked second (AUC = 0.679, 95% CI: 0.635–0.723; cutoff ≥ 2433.85). The MLR (AUC = 0.658, 95% CI: 0.612–0.702; cutoff ≥ 0.645) and NLR (AUC = 0.636, 95% CI: 0.589–0.680; cutoff ≥ 10.15) followed, demonstrating moderate diagnostic utility. The PLR (AUC = 0.638, 95% CI: 0.592–0.682; cutoff ≥ 224.38) and NMR (AUC = 0.638, 95% CI: 0.592–0.682; cutoff ≥ 6.38) showed comparable performance, while the NPR had the lowest diagnostic accuracy (AUC = 0.567, 95% CI: 0.521–0.613; cutoff ≥ 0.0405) (Figure 2). Detailed diagnostic metrics, including sensitivity, specificity, PPV, and NPV are presented in Table 2.

Multivariate logistic regression identified CLR > 11.77 (OR 4.06, 95% CI 2.71–6.08, *p* = 0.001), PIV > 2433.85 (OR 2.17, 95% CI 1.50–3.14, *p* < 0.001), temperature (OR 1.60 95% CI 1.17–2.18 per 1 °C increase), and younger age as independent predictors. (OR 0.93 (95% CI 0.89–0.98 per additional year) (Table 3).

Ultrasound visualized the appendix in 656/878 (74.7%) cases. Positive (i.e., consistent with appendicitis) findings occurred in 523/696 (75.1%) simple cases and 109/182 (59.9%) complicated cases. Non-visualization was more frequent in patients with CA (59/182, 32.4%) than in those with SA (140/696, 20.1%). Among visualized complicated cases, sensitivity reached 94.0% (109/116) while sensitivity among visualized SA was 96.9% (523/540).

## 4. Discussion

Despite advances in diagnostic modalities, the initial misdiagnosis rate for appendicitis in children remains high, and ranges from 28% to 57% for children 12 years old or younger to nearly 100% in those 2 years old or younger [18]. Earlier research indicates that 5.9% to 27.6% of acute appendicitis cases involved a missed diagnosis, which subsequently led to a rise in perforation rates from a baseline of 20.3–28.0% to as high as 33.3–50.0% [19,20].

Traditional diagnostic approaches include clinical scoring systems such as the Pediatric Appendicitis Score (PAS), laboratory markers such as WBC count and CRP, and imaging modalities such as ultrasound and CT. However, each of these measures has notable limitations. The PAS and Alvarado scores are sometimes used to assess the likelihood of acute appendicitis in children, with scores above 7 indicating a high probability [21,22]. However, in a study evaluating PAS in children, it was found that the score had low sensitivity in both younger and older children, with significantly lower sensitivity in those under 4 years old, leading to delays in diagnosis and increased complications [23]. Moreover, the ability of the PAS and Alvarado scores to differentiate between SA and CA is limited. Studies have demonstrated that neither score reliably predicts disease severity, often requiring intraoperative findings or advanced imaging for definitive classification [24].

CRP is a reliable marker for distinguishing CA from SA in children. A cutoff of 4.3 mg/dL yielded 74% sensitivity and 77% specificity, while CRP ≥ 10 mg/L demonstrated 87% sensitivity and 77% specificity for appendicitis overall [25,26]. In some studies, all children with perforations had a CRP > 20 mg/L (median 96 mg/L, compared to 5 mg/L in non-appendicitis cases). Elevated CRP also correlated with longer hospital stays [27,28]. However, in the case of SA, 30% of children had normal CRP and 33.6% had normal WBC, limiting its standalone utility. Therefore, combining CRP levels with other measures is beneficial. For example, combining CRP > 40 mg/L with PAS ≥ 8 improved specificity for CA to over 90% [29].

Ultrasound is the imaging modality of choice for the diagnosis of appendicitis in children due to its ability to provide assessments without exposing children to ionizing radiation. The “as low as reasonably achievable” (ALARA) principle limits the use of CT for the diagnosis of appendicitis and prioritizes ultrasound to minimize radiation exposure. However, the accuracy of ultrasound for the diagnosis of appendicitis is highly operator-dependent and can vary widely. Its moderate sensitivity (33.9–51.5%) for CA underscores the necessity for complementary diagnostic tools, such as blood biomarkers [30,31]. Blood biomarkers such as CRP and WBC can provide additional diagnostic clues, reducing the need for CT imaging in low-risk patients while maintaining accuracy [32,33].

This study demonstrates that preoperative inflammatory biomarkers, particularly CLR, offer valuable diagnostic utility in differentiating complicated from SA in pediatric patients. The significant elevations in NLR, MLR, PLR, NMR, NPR, PIV, and CLR in the CA group reflect a heightened systemic inflammatory response associated with disease severity. CLR emerged as the most effective biomarker, with an AUC of 0.772 and odds ratio of 5.935 (*p* < 0.001) for CA at a cutoff of ≥11.77. Our findings are in line with a smaller pediatric study, which reported good CLR performance (AUC 0.89; however, it is not possible to compare cutoff values between our study and this previous study as CRP normal range was not reported). In the current study, CA was defined based on histopathology readings, and a comparison was made between perforated (237 cases) and non-perforated appendicitis (69 cases) [13]. This grouping excluded gangrenous appendicitis and localized abscesses—common complicated forms in children, which should be treated like perforated appendicitis. Similar results emerged from an even smaller adult study, which compared perforated appendicitis (111 cases) and acute appendicitis (20 cases) based on pathology reports, and found the highest AUC for CLR (0.83). It is not possible to compare cutoff values between our study and this previous study as CRP normal range was not reported [14]. In our larger cohort (n = 878) using the broader AAST grading (grades ≥ 2), CLR maintained strong accuracy (AUC 0.772), confirming its reliability across the full spectrum of complicated disease rather than perforation alone. In another pediatric study, CLR was evaluated in acute appendicitis in cases associated with Enterobius versus cases without Enterobius (430 total cases). Unfortunately, this study did not compare cases with inflammation to cases without inflammation [34].

Notably, the performance of CLR surpasses that of previously reported hematologic ratios in pediatric cohorts. In Ha et al. (2024) [35], NLR (AUC 0.653, *p* = 0.003), PLR (0.644, *p* = 0.004), and MLR (0.622, *p* = 0.016) demonstrated only moderate discrimination, while Tekeli et al. (2023) [36], reported similar modest results for NLR (AUC 0.621, *p* < 0.001) and PLR (0.624, *p* < 0.001). Taken together, these findings underscore CLR’s superior diagnostic accuracy in identifying CA in children. In our study, PIV, which integrates neutrophils, monocytes, and platelets relative to lymphocyte counts, emerged second in its effectiveness, with an AUC of 0.679 and OR of 3.348 (*p* < 0.001) for CA at a cutoff of ≥2433.85. This composite index likely captures a broader spectrum of the inflammatory cascade, enhancing its discriminatory power compared to individual ratios such as NLR or MLR. The superior performance of PIV suggests that combining multiple inflammatory markers may better reflect the complex pathophysiology of complicated appendicitis, including neutrophilia, monocytosis, thrombocytosis, and reduced lymphocyte counts. NLR, a well-established marker in appendicitis, demonstrated a significant association with CA, aligning with previous studies that reported elevated NLR in cases of gangrenous or perforated appendicitis [37,38]. In this study, an NLR cutoff of ≥10.15 was associated with a 2.45-fold increased likelihood of CA, reinforcing its role in early risk stratification. Similarly, MLR, though less frequently studied in pediatric appendicitis, demonstrated promising diagnostic value, with a cutoff of ≥0.645 yielding a 2.78-fold increased odds of CA. This finding suggests that monocytic activation, possibly driven by tissue necrosis or abscess formation, may play a critical role in severe cases [35,39,40]. PLR and NMR also exhibited statistically significant predictive capacity, though their performance was slightly inferior to CLR, PIV, and MLR. NPR demonstrated the least discriminatory power, suggesting it may be more suitable as a supplementary rather than primary diagnostic tool. Elevated CRP levels were demonstrated in the CA group (median 57.11 mg/L vs. 11.20 mg/L in SA). This is in accordance with previous data, which report CRP to be a sensitive and reliable factor for appendicular perforation, particularly at levels > 40 mg/L when combined with clinical scores such as appendicitis inflammatory response (AIR) score or PAS [27,41,42,43].

We think that CLR outperformed other ratios because it directly integrates the intense acute-phase response (markedly elevated CRP in perforation/gangrene due to bacterial translocation and necrosis) with stress-induced lymphopenia—two hallmarks of complicated disease. In contrast, NLR/PLR/MLR rely mainly on relative shifts in leukocytes/platelets that occur in both simple and complicated cases, while PIV dilutes the dominant CRP signal. This explains CLR’s highest AUC (0.772) and strongest association (OR 5.935) in our cohort.

The observed temperature difference (37.27 °C vs. 36.98 °C), though statistically significant, is likely clinically irrelevant. Studies demonstrate that fever may increase the Likelihood Ratio (LR~3.4) of appendicitis, but only at higher thresholds (≥38 °C), thus limiting the utility of minor temperature changes [32,33].

The multivariate logistic regression analysis (Table 3) identified several key independent predictors of CA in pediatric patients, including CLR (>11.77), PIV (>2433.85), temperature, and younger age. Specifically, CLR and PIV demonstrated strong associations with complicated appendicitis, with odds ratios of 4.06 and 2.174, respectively, highlighting their potential as valuable biomarkers in the diagnostic process. Younger age and temperature further reinforced their relevance, supporting the utility of combining these clinical and laboratory markers to improve early risk stratification and guide treatment decisions. Importantly, CLR and PIV should not be viewed as competing markers. Rather, their simultaneous significance in multivariable analysis suggests that CRP-driven and cell-mediated inflammatory responses provide complementary information regarding disease severity.

Ultrasound demonstrated high sensitivity for both SA and CA when the appendix was visualized. However, the appendix was often not visualized, which limits its discriminatory power compared to blood-based biomarkers.

Our findings have the potential to assist the clinician with management decisions in equivocal cases. As urgent surgery is advised in cases of CA (unless a significant abscess is diagnosed) in contradistinction to the option of delaying the operation up to 24 h and even trying non-operative treatment with acute appendicitis, the distinction between simple and CA is crucial to decision making. Of note, we included gangrenous appendicitis in our CA group despite the fact that, in some studies, it is not always treated as CA to the same extent as perforated/abscess cases, but mostly refers to the extent of post-operative complications and post-operative antibiotic treatment [44,45]. However, we think that non-operative treatment and delaying surgery should not be routinely offered when gangrenous appendicitis is suspected, as data points to higher failure rates and increased complications with non-operative treatment [46,47]. Therefore, we included gangrenous appendicitis in the group.

The retrospective design and single-center setting of the study harbor limitations, potentially affecting generalizability. The single-center, 6-year design may also introduce minor intra-laboratory variability (e.g., reagent/calibration changes), despite uniform use of the Sysmex XN-1000, Kobe, Japan. Therefore, multicenter validation is needed to confirm CLR reproducibility. In addition, in our study we did not calculate time elapsed between symptom onset to blood draws, and between blood draws to surgery. The variability in timing may confound biomarker performance, especially because CRP, which is highly time dependent, is a significant component of CLR, which was found to be the best predictor of complicated appendicitis. The later occurrence of CA in the course of disease [48] may mitigate this confounding effect. Additionally, other clinical data that were not analyzed in this study, such as comorbidities, prior antibiotic therapy, and vital signs other than temperature, may also affect biomarker values and limit generalizability of the results.

Future prospective, multicenter studies are warranted to validate these findings, establish standardized clinical thresholds, and assess the integration of these biomarkers into routine diagnostic algorithms or clinical scoring systems.

## 5. Conclusions

In conclusion, CLR, alongside PIV, NLR, MLR, PLR, and NMR, provides a valuable, accessible means of stratifying management-oriented and clinically relevant pediatric appendicitis severity. These biomarkers are not intended to replace currently used measures but to serve as a simple, cost-free adjunct, which may assist clinical decision making. The biomarkers, particularly CLR, hold promise for improving diagnostic accuracy and guiding treatment decisions, especially in resource-limited settings.

## Figures and Tables

**Table 1:** Comparison of Complicated and Simple Appendicitis.

		Complicated Appendicitis (*n* = 182)	Simple Appendicitis (*n* = 696)	*p*-Value
Sex	Male/Female	110 Male 72 Female	466 Male 230 Female	0.11
Age, years	Mean (SD) Median (95% CI)	10.0 (4.26) 9.0 (9.32–10.57)	11.8 (3.71) 12.0 (11.53–12.08)	<0.001 *
Neutrophils (K/μL)	Mean (SD) Median (95% CI)	13.61 (4.92) 13.55 (12.90–14.20)	11.39 (4.29) 11.20 (11.00–11.60)	<0.001
Lymphocytes (K/μL)	Mean (SD) Median (95% CI)	1.50 (0.86) 1.30 (1.20–1.50)	1.74 (0.85) 1.60 (1.50–1.70)	<0.001 *
Monocytes (K/μL)	Mean (SD) Median (95% CI)	1.23 (1.41) 1.10 (1.10–1.20)	0.95 (0.48) 0.90 (0.90–1.00)	<0.001 *
Platelets (K/μL)	Mean (SD) Median (95% CI)	294.31 (72.73) 283 (270–301)	270.15 (72.08) 261 (257–268)	0.031 *
CRP (mg/L)	Mean (SD) Median (95% CI)	88.55 (97.75) 57.11 (44.53–66.92)	27.15 (44.74) 11.20 (9.29–13.27)	0.001 *
Temp (deg C)	Mean (SD) Median (95% CI)	37.27 (0.74) 37.05 (37.00–37.10)	36.98 (0.45) 36.90 (36.90–37.00)	<0.001 *
NLR	Mean (SD) Median (95% CI)	12.19 (7.44) 10.53 (9.00–11.83)	8.89 (7.21) 6.93 (6.47–7.50)	<0.001 *
MLR	Mean (SD) Median (95% CI)	1.02 (1.33) 0.82 (0.73–0.89)	0.68 (0.48) 0.57 (0.53–0.60)	<0.001 *
PLR	Mean (SD) Median (95% CI)	254.89 (147.46) 224.38 (202.30–237.50)	194.94 (117.50) 163.89 (172.27–155.45)	<0.001 *
NMR	Mean (SD) Median (95% CI)	12.23 (8.48) 10.53 (9.00–11.83)	8.90 (7.01) 6.93 (6.47–7.50)	<0.001*
NPR	Mean (SD) Median (95% CI)	0.05 (0.02) 0.05 (0.04–0.05)	0.04 (0.02) 0.04 (0.04–0.04)	0.004 *
PIV	Mean (SD) Median (95% CI)	4558.18 (7912.10) 3192.40 (2758.20–3799.60)	2393.29 (2360.44) 1600 (1495.70–1837.50)	<0.001 *
CLR	Mean (SD) Median (95% CI)	81.73 (261.41) 41.88 (30.00–50.98)	20.03 (45.07) 7.09 (5.87–8.24)	<0.001 *

* The Mann–Whitney U Test for a non-parametric test was performed. Abbreviations: CLR, CRP-to-lymphocytes ratio; CRP, C-Reactive-protein; MLR, monocytes-to-lymphocytes ratio; NLR, neutrophils-to-lymphocytes ratio; NMR, neutrophils-to-monocytes ratio; NPR, neutrophils-to-platelet ratio; PIV, pan-immune inflammation value; PLR, platelet-to-lymphocytes ratio.

**Table 2:** ROC Analysis.

Biomarker	Cutoff	Area (AUC) 95% CI (Lower-Upper)	*p*-Value	Sensitivity (%) 95% CI (Lower-Upper)	Specificity (%) 95% CI (Lower-Upper)	PPV (%)	NPV (%)
NLR	≥10.15	0.636 (0.589–0.680)	*p* < 0.001	52.5 (44.9–59.5)	69.2 (65.8–72.6)	30.8	85.0
MLR	≥0.65	0.658 (0.612–0.702)	*p* < 0.001	65.9 (58.8–72.4)	58.9 (55.3–62.5)	29.5	87.1
PLR	≥224.38	0.638 (0.592–0.682)	*p* < 0.001	50.0 (42.8–57.2)	71.3 (68.0–74.7)	31.3	84.8
NMR	≥6.38	0.638 (0.592–0.682)	*p* < 0.001	73.6 (66.8–79.5)	45.5 (42.0–49.3)	26.1	87.1
NPR	≥0.04	0.567 (0.521–0.613)	*p* = 0.005	70.3 (63.3–76.5)	43.9 (40.5–47.9)	24.7	85.2
PIV	≥2433.85	0.679 (0.635–0.723)	*p* < 0.001	63.7 (56.5–70.4)	65.1 (62.0–69.0)	32.3	87.3
CLR	≥11.77	0.772 (0.733–0.812)	*p* < 0.001	78.0 (71.5–83.4)	62.6 (58.9–66.1)	35.3	91.6

Abbreviations: CLR, CRP-to-lymphocytes ratio; CRP, C-Reactive-protein; MLR, monocytes-to-lymphocytes ratio; NLR, neutrophils-to-lymphocytes ratio; NMR: neutrophils-to-monocytes ratio; NPR: neutrophils-to-platelet ratio; PIV, pan-immune inflammation value; PLR, platelet-to-lymphocytes ratio.

**Table 3:** Multivariate Logistic regression: Independent Predictors of Complicated Appendicitis.

Variable	B	S.E.	Wald	*p*-Value	Odds Ratio	95% Confidence Interval
CLR (>11.77)	1.401	0.206	46.306	<0.001	4.06	2.71–6.08
PIV (>2433.85)	0.776	0.188	17.035	<0.001	2.17	1.50–3.14
Age (years)	−0.07	0.024	8.624	0.003	0.93	0.89–0.98
Temperature (°C)	0.468	0.158	8.758	0.003	1.60	1.17–2.18
Constant	−19.15	5.904	10.515	0.001	0.00	-

Model adjusted for all listed variables (Enter method), statistical significance was assessed based on the Wald test; Statistically significant results are indicated by *p*-values less than 0.05; B = Regression coefficient; S.E. = Standard error of the regression coefficient. Age is continuous (negative coefficient indicates higher risk in younger children); CLR and PIV are binary (CLR > 11.77, PIV > 2433.85); temperature is a continuous variable. Abbreviations: CLR, CRP-to-lymphocytes ratio; CRP; PIV, pan-immune inflammation value.

## Data Availability

The data presented in this study are available on request from the corresponding author due to IRB and hospital policy.

## Abbreviations

The following abbreviations are used in this manuscript:

AAST	American Association for the Surgery of Trauma
ALARA	As Low As Reasonably Achievable
AUC	Area Under the Curve
CA	Complicated Appendicitis
CBC	Complete Blood Count
CLR	CRP-to-Lymphocytes Ratio
CRP	C-Reactive protein
MLR	Monocytes-to-Lymphocytes Ratio
NLR	Neutrophil-to-Lymphocytes Ratio
NMR	Neutrophil-to-Monocyte Ratio
NPR	Neutrophil-to-Platelet Ratio
OR	Odds Ratio
PAS	Pediatric Appendicitis Score
PIV	Pan-Immune-Inflammation Value
PLR	Platelets-to-Lymphocytes Ratio
SA	Simple Appendicitis
SIRS	Systemic inflammatory response syndrome
